# Supplementary material for: Identification of photoperiod-induced specific miRNAs in the adrenal glands of Sunite sheep (Ovis aries)
Source: Front Vet Sci. 2022 Jul 22;9:888207. doi: 10.3389/fvets.2022.888207 (PMC9354845; doi:10.3389/fvets.2022.888207)
Supplement: Supplementary file 1 [file Data_Sheet_1.ZIP › Supplementary file/miRDeep2 file.zip!/miRDeep2 file/Novel_Structure/LP42A3/Novel_541.pdf]

Provisional ID	:	NC_019467.2_12349
Score total	:	12.8
Score for star read(s)	:	3.9
Score for read counts	:	5.2
Score for mfe	:	2
Score for randfold	:	1.6
Score for cons. seed	:	
Total read count	:	22
Mature read count	:	15
Loop read count	:	0
Star read count	:	7

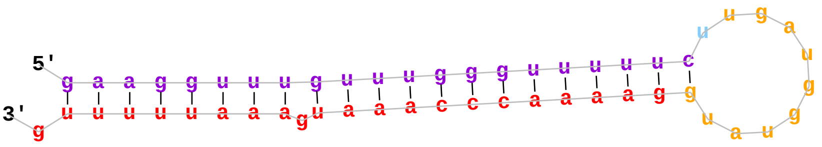

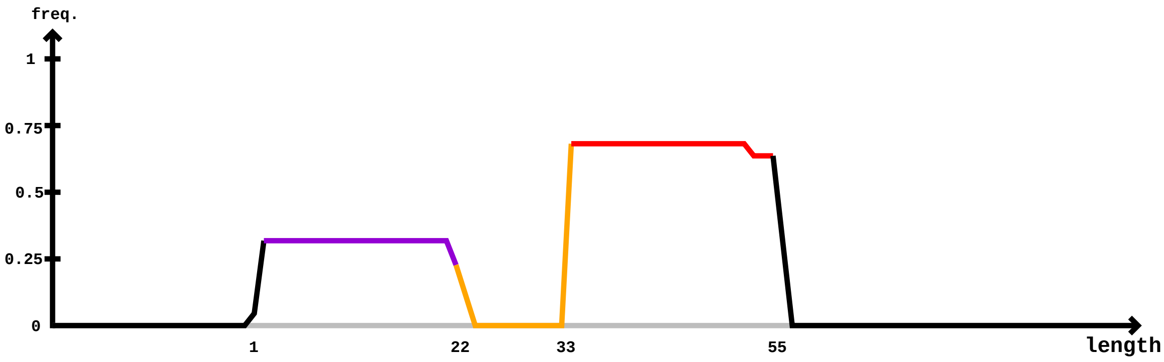


```
                    Star                         Mature
5'- guaaauuauuggguuggccagaagguuuguuuggguuuuucuugaugguauggaaaacccaaaugaaauuuuuggccaaccuaauacuuaauuucucagucuguucauucc   -3'   obs
    guaaauuauuggguuggccagaagguuuguuuggguuuuucuugaugguauggaaaacccaaaugaaauuuuuggccaaccuaauacuuaauuucucagucuguucauucc         exp
    ......((((((((((((((((((.((((((((((((((((..........)))))))))))))))).)))))))))))))))))).........................   reads    mm    sample
    ...................aAaagguuuguuuggguuuuuc......................................................................   1        1     seq
    ....................Aaagguuuguuuggguuuuu.......................................................................   1        1     seq
    ....................gaaAguuuguuuggguuuuu.......................................................................   1        1     seq
    ....................gaagguuuguuuggguuuuuc......................................................................   1        0     seq
    ....................gaaAguuuguuuggguuuuuc......................................................................   2        1     seq
    ....................Aaagguuuguuuggguuuuuc......................................................................   1        1     seq
    ....................................................gaaaacccaaaugaaCuuu........................................   1        1     seq
    ....................................................gaaaacccaaaugaaCuuuuug.....................................   14       1     seq
```
